# Meningoencephalitis and retinal vasculitis due to rickettsial infection

**DOI:** 10.1007/s00415-023-12097-z

**Published:** 2023-11-24

**Authors:** Louisa Lehner, Stephan Thurau, Konstantin Pusl, Steffen Tiedt, Florian Schöberl, Robert Forbrig, Günter Höglinger, Michael Strupp

**Affiliations:** 1 Department of Neurology, LMU University Hospital, Ludwig Maximilians University Munich Marchioninistr, 15, 81377 Munich, Germany; 2 Department of Ophthalmology, LMU University Hospital, LMU Munich, Munich, Germany; 3 Institute for Stroke and Dementia Research (ISD), LMU University Hospital, LMU Munich, Munich, Germany; 4 Institute of Neuroradiology, LMU University Hospital, LMU Munich, Munich, Germany

Dear Sirs,

Rickettsia are obligate intracellular bacteria with a wide geographic distribution throughout the world. They are transmitted via ectoparasites such as ticks, fleas, mites, or lice [1]. Rickettsioses are divided into four main groups: the classical spotted fever group (typhus group, for example Rickettsia prowazekii), the tick-bite fever group (rocky mountain spotted fever group (RMSF), best known representative Rickettsia rickettsia), the Tsutsugamushi fever group (scrub typhus, Orientia tsutsugamushi) and a transitional group [2]. The clinical presentation can be very heterogeneous depending on the pathogen. The main symptoms are flu-like, including high fever, headache, aching limbs, nausea and vomiting. Individual subgroups may cause typical exanthema. As rickettsia have a tropism for vascular endothelial cells, they can cause direct vascular injury, resulting in a generalized vasculitis, which can manifest in any organ [3]. Here we present a patient with a distinct central nervous system manifestation due to rickettsia infection.

Case report. Three weeks after returning from a one-month trip to Mexico, a 23-year-old student developed unspecific chest pain. Over the next few days, she also experienced abdominal pain and shortness of breath. An outpatient electrocardiogram, laboratory tests including cardiac enzymes and abdominal ultrasound were without pathological findings. She had no medical history and was not taking any medication. Apart from a brief episode of diarrhea, there were no particular events during her trip.

She presented to our emergency department with increasing pain exacerbation and reported especially severe pain in the legs and an intense headache. During the examination, a meningism was noticed, so a lumbar puncture was performed, which revealed a lympho-monocytic pleocytosis (23 cells/µl; upper limit of normal range: 5/µl) with normal glucose and protein levels. The multiplex-PCR panel for meningitis/encephalitis was negative. Because infectious meningitis with an unknown pathogen was suspected, treatment with intravenous ceftriaxone and acyclovir was initiated. A brain MRI, performed on day 3 of admission, revealed circular parenchymal lesions at the supratentorial gray-white junction (both in parafalcin and convexity location), partially contrast-enhancing, located in both hemispheres (Fig. 1A).

**Fig. 1:**
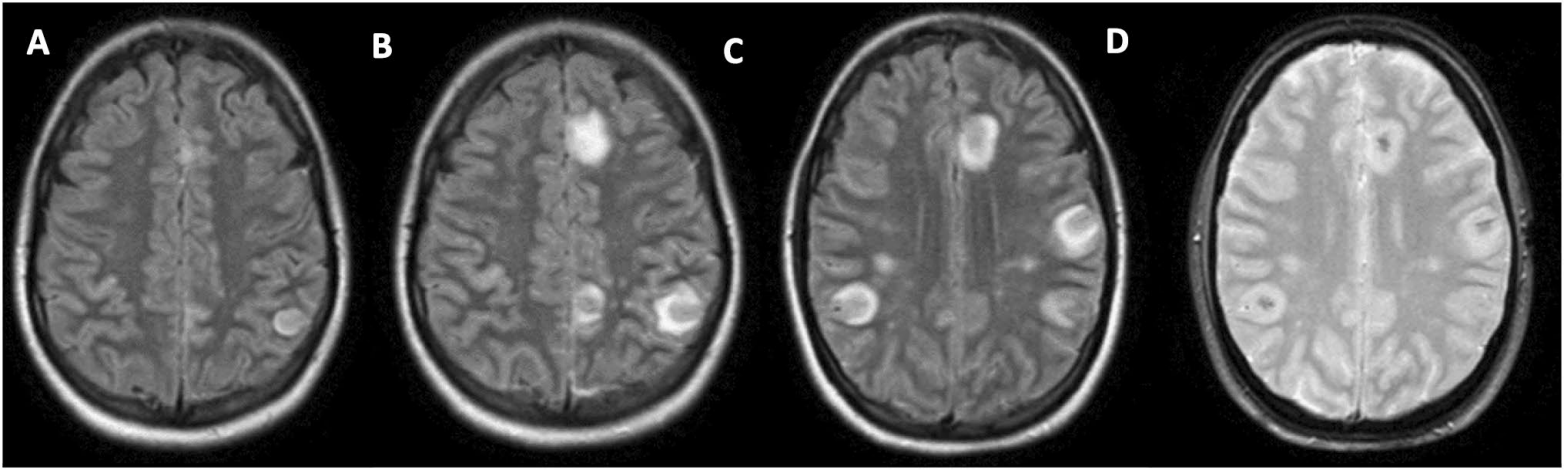
Brain MRI. Brain MRI 1 week after symptom onset showed multiple T2-FLAIR hyperintense lesions particularly located at the supratentorial gray-white junction (**A**). MRI 3 days later revealed a massive worsening of the imaging findings with an increase of the lesions both in size and number (**B**, **C**), now also showing central hemorrhagic transformation (**D**, T2*: dark intralesional spots)

Within 2 days, her clinical condition worsened with high fever, unresponsive to standard antipyretic medication, vomiting and intermittent reduced vigilance: a second cerebrospinal fluid (CSF) analysis showed an increased pleocytosis with 229 cells/µl and elevated protein (145 mg/dl). The MRI performed on day 6 after admission showed a massive progression of the intracranial lesions in terms of size and number (Fig. 1B, C). The T2*/gradient echo sequences revealed a central hemorrhage within the lesions (Fig. 1D). Consequently, polypragmatic antibiotic treatment was escalated to intravenous meropenem and vancomycin; acyclovir was continued. There was a positive treatment response with a decrease of the pleocytosis and protein levels (142 cells/µl, 177 mg/dl) and stable MRI-lesions on day 7 of admission. To cover atypical pathogens, doxycycline 200 mg was added to the antibiotic regimen on day 10 of admission.

Until day 17 the following CSF-tests were negative: CSF-PCR-panel including Escherichia coli, Haemophilus influenzae, Listeria monocytogenes, Neisseria meningitidis, Streptococcus agalactiae and pneumoniae, Cytomegalovirus (CMV), Epstein-Barr virus (EBV), Herpes-simplex virus (HSV) 1, 2 and 6, Varicella-zoster virus (VZV), and Enterovirus. Additionally, the following CSF-PCR were performed with a negative result: Cryptococcus neoformans/gattii, universal fungal PCR, Tropheryma whipplei, Toxoplasma gondii, Naegleria fowler, Acanthamoeba, Aspergillus, Adenovirus, Leptospira. The antibody-CSF-serum-index for Borrelia burgdorferi, VZV, Measles virus and Rubella virus were negative.

Blood cultures were negative. There was immunity for Hepatitis A; Hbs-antigen, Anti-Hbc-IgG/IgM, Anti-HCV-IgG and Anti-HIV1/2 + p24-Antigen were negative. IgG and IgM antibodies for Chikungunya virus, Dengue virus and West Nile virus were negative. Microscopic examination of blood smear showed a negative result of malaria. The following laboratory tests also remained negative: Candida antigen, CMV, Coxsackie virus, Cryptococcus antigen, Entamoeba hystolitica, Echinococcus, Histoplasma, Leptospira, Spirochetes coccoides, Tick borne encephalitis virus, Treponema pallidum, Toxoplasmosis, Trypanosoma brucei und cruzei. PCR of Sars-CoV-2, Influenza A and B were negative. The serological vasculitis parameters were unremarkable except for a minimally elevated titer of antinuclear antibodies (1:200) with unremarkable ANA-subdifferentiation.

Based on the MRI findings, acute disseminated encephalomyelitis (ADEM) was discussed as a differential diagnosis to infectious genesis. Initially, a stereotactic biopsy was considered, but since the patient improved clinically as well as in terms of CSF diagnostics and imaging under the existing therapy, brain biopsy was not performed.

Ultimately, we received a positive serum result for rickettsia. There was a positive titer for rickettsia of the tick-bite fever group (IgM 1:128, IgG 1:32; normal values IgM < 1:32, IgG < 1:32) and the Tsutsugamushi group (IgM 1:128, IgG negative; normal values IgM < 1:64, IgG < 1:128). Regarding the spotted fever group, only IgG antibodies were weakly positive without IgM antibodies. Rickettsia PCR could not be performed in the absence of skin lesions for sample collection. Additionally, an increase of borrelia-IgM-antibodies in the second CSF-sample was noted. We attributed this to a cross-reaction in the antigen search test.

Therefore, the final diagnosis of rickettsia infection with meningoencephalitis and vasculitis was made. Therapy was changed accordingly to monotherapy with doxycycline 200 mg once daily and administered for 21 days. Clinically, there was a marked and rapid improvement. A decrease in the cell count to 34/µl was observed in the CSF on day 14 of admission.

On day 14 of admission and day 4 of doxycycline therapy, the patient reported new black spots in her visual fields of both eyes. The visual acuity was reduced to 0.1 in the right and 0.4 in the left eye. Ophthalmologic examination revealed extensive findings of arterial and venous vascular occlusion, retinal hemorrhages, and cotton wool spots (Fig. 2). Assuming a peri-infectious vasculitic autoimmune etiology, we started therapy with oral prednisolone 100 mg for three days with a subsequent tapering regimen. Initially, this showed a stabilization but no significant improvement. In a follow-up examination 4 weeks after completion of antibiotic treatment, the intracerebral lesions were regressive, and the CSF cell count had normalized. However, the visual disturbances worsened again secondarily, so that a steroid pulse therapy (500 mg intravenous for 3 days) was necessary to maintain the vision at a stable, but poor level (visual acuity right eye 0,25, left eye 0,63). Due to the relapse, a permanent steroid-sparing therapy with a tumour necrosis factor (TNF)-α blocker (adalimumab) every 2 weeks and 10 mg of methotrexate once a week was started.

**Fig. 2:**
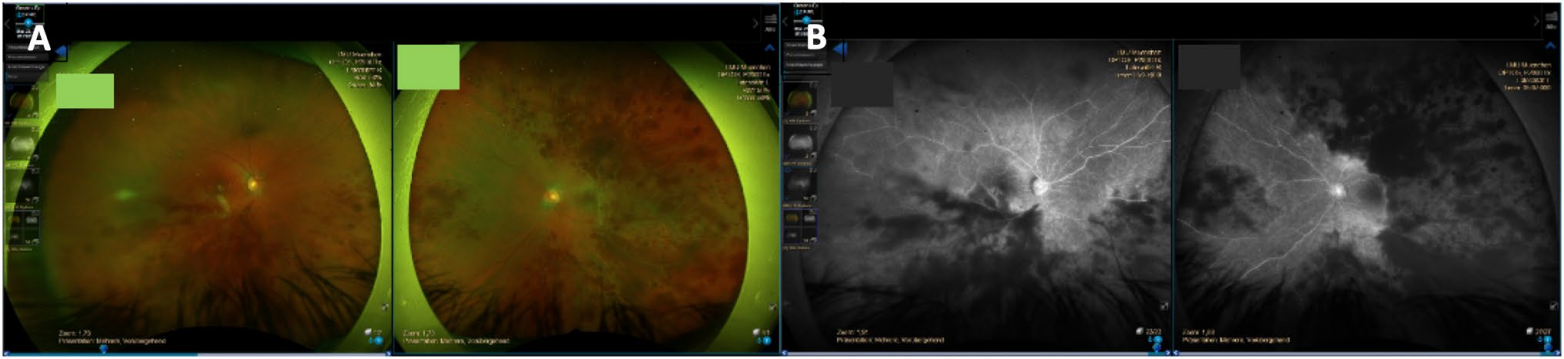
Funduscopic examination and fluorescein angiography. Fundoscopy revealed retinal arterial and venous vascular occlusions, hemorrhages around the occlusions and cotton wool spots (**A**). Fluorescein angiography showed hemorrhages and a lack of perfusion of retinal arteries (**B**)

Rickettsioses are rare in Europe but widely distributed throughout the USA, Central and South America as well as Asia. Both the clinical and serological diagnosis of rickettsial infection is challenging. The most widely used diagnostic method includes serological detection methods, which, however, can only provide positive evidence from week two at the earliest [4]. Due to the close antigen relationship, there is a high degree of cross-reactivity within the members of the rickettsiae group [5]. Because of the geographic distribution of the tick-bite fever group/RMSF-group in Mexico [6] and the positive IgG (1:32) and IgM (1:128) antibodies, this group was suspected as the causative pathogen in our patient. Typically, patients with rickettsia infection have a history of a tick bite, a rash is present in up to 90% of the patients and may facilitate the diagnosis [7, 8]. Our patient did not have any skin lesions but very unspecific symptoms including high fever, headache, myalgias, abdominal pain, nausea and vomiting, which can occur not only with rickettsiae but also with other bacterial and viral infections. MRI findings of multiple intracranial, partly contrast-enhancing, lesions with central hemorrhage are rarely documented to the extent that they were in our patient's case. More common MRI findings in RMSF include meningeal enhancement and periventricular subcortical infarctions, secondary to vasculitis [9, 10].

Another particularity in the case of our patient were the ophthalmologic complications which occurred only secondarily. Ocular involvement with retinitis and retinal vasculitis are common findings in RMFS patients, however they usually occur with the initial symptoms and respond well to therapy [11]. Pathophysiologically, ocular damage may be due to two mechanisms: on the one hand, a direct invasion of the retinal vessels by the bacteria and, on the other hand, as a secondary immunological response to the bacterium [12]. Considering the delayed onset of ophthalmologic symptoms despite adequate antibiotic therapy, we assumed a secondary immunological etiology in our patient and therefore decided to start the oral cortisone therapy with a subsequent tapering regimen and a steroid-sparing therapy.

In summary, rickettsial infection is an important differential diagnosis in patients with meningoencephalitic syndrome after travel to the USA, South America, and Asia. The most challenging aspect of this particular case was the identification of the causative pathogen. Unbiased diagnostic tools such as metagenomic next-generation sequencing, which is becoming more and more accessible, might have helped to identify the pathogen more rapidly [13]. Vasculitic complications are common but rarely documented to the extent reported in our patient [14, 15]. Consistent with other case series, the initial response to antibiotic therapy was good. Whether the secondary retinal vasculitic complications could have been avoided by starting antibiotic therapy even earlier or with early on additional steroids remains to be discussed.

## Data Availability

All relevant data can be found in the article. The participant of this study did not give written consent for further data to be shared publicly, so due to the sensitive nature of the research further supporting data is not available.
